# The etiology of extensive pleural effusions with troublesome clinical course among children

**DOI:** 10.1590/S1516-31802004000600008

**Published:** 2004-11-04

**Authors:** Luís Marcelo Inaco Cirino, Filumena Maria da Silva Gomes, Bernardo Nogueira Batista

**Keywords:** Pleural empyema, Pneumonia, Child, Pleural effusion, Epidemiology, Empiema pleural, Pneumonia, Criança, Derrame pleural, Epidemiologia

## Abstract

**CONTEXT:**

In São Paulo, pneumonia is the main infectious cause of death among children. Parapneumonic pleural effusion is a possible complication and has to be treated surgically when the patient does not respond to antibiotics.

**OBJECTIVE:**

Assessment of the etiology of complicated parapneumonic pleural effusions that needed surgical intervention.

**TYPE OF STUDY:**

Retrospective study.

**SETTING:**

University hospital of the University of São Paulo.

**METHOD:**

Analysis of 4,000 files on children hospitalized with pneumonia from November 1986 to November 1996 had shown that 115 of these children presented a total of 117 cases of pleural empyema that required surgical procedures. The children's clinical condition was assessed in relation to radiological findings and to their nutrition and immunization status. Previous antimicrobial therapy and pleural effusion bacterioscopy were also evaluated.

**RESULTS:**

*Streptococcus pneumoniae* was the agent found most commonly, as frequently in blood cultures as in pleural effusions.

**DISCUSSION:**

Data on vaccination coverage, birth weight and nutritional status are analyzed and compared to other publications. We observed that pleural effusion has a high potential for discomfort, and in most cases it is not a complication of the first pulmonary disease episode. Previous use of antibiotics interfered with culture positivity. The agent most frequently found was *Streptococcus pneumoniae*, which is in accordance with the findings from other authors. Nonetheless, the antibiotics used to treat the patients after the procedure were the same used in non-complicated pneumonias, which has led us to conclude that the worse outcome in this cases was not due to drug resistance.

**CONCLUSION:**

The bacteriological profile in our series of complicated pneumonia cases was similar to what has been described for non-complicated pneumonia cases. Future studies will be necessary to determine why these children presented a worse outcome.

## RESUMO

**CONTEXTO:**

Em São Paulo, pneumonia é a principal causa infecciosa de morte infantil. Derrame pleural parapneumônico é uma complicação grave da doença e intervenção cirúrgica pode ser necessária quando o paciente não responde à antibioticoterapia.

**OBJETIVO:**

Determinar a etiologia dos derrames pleurais parapneumônicos complicados que necessitaram de intervenção cirúrgica.

**TIPO DE ESTUDO:**

Retrospectivo.

**LOCAL:**

Hospital Universitário da Universidade de São Paulo.

**MÉTODOS:**

A análise de 4.000 prontuários de crianças hospitalizadas por pneumonia de novembro de 1986 a novembro de 1996 mostrou que 115 crianças apresentaram um total de 117 casos de empiema que necessitaram de intervenção cirúrgica. Os autores analisaram os dados clínicos das crianças, correlacionando os achados radiológicos, o estado nutricional e a situação vacinal das crianças. Terapias antimicrobianas prévias e bacterioscopia do derrame pleural também foram analisadas.

**RESULTADOS:**

*Streptococcus pneumoniae* foi o agente mais encontrado, tanto nas hemoculturas quanto nos derrames pleurais.

**DISCUSSÃO:**

A cobertura vacinal, o peso ao nascimento e o estado nutricional das crianças estudadas foram analisados e comparados aos dados encontrados em outras publicações. Observamos que derrames pleurais causam desconforto importante e, na maioria dos casos, não se trata de complicação do primeiro episódio infeccioso do aparelho respiratório. O uso prévio de antibióticos alterou o resultado das culturas. O achado de *Streptococcus pneumoniae* como o agente mais freqüentemente encontrado está de acordo com os resultados de outros autores. No entanto, os antibióticos utilizados após a realização do procedimento são os mesmos utilizados em pneumonias não-complicadas, fato que nos levou a concluir que a pior evolução desses casos não se deve à resistência dos agentes aos antimicrobianos.

**CONCLUSÃO:**

O perfil bacteriano na nossa série de casos é semelhante ao descrito para pneumonias não complicadas. Novos estudos serão necessários para se determinar o motivo da pior evolução dessas crianças.

## INTRODUCTION

Pneumonia is the main cause of death in children in São Paulo city, among deaths due to infectious diseases.^1^ The majority of such patients are treated empirically, without etiological confirmation, on an outpatient basis. The causative infectious agent in hospitalized pneumonia patients can be isolated in 60% of the cases.^2^

Many different specimens can be cultured with the aim of the diagnosing the etiological agent in pneumonia: material obtained through thoracocentesis directed towards the pneumonic focus; oropharyngeal swab; blood and cricothyroid transmembrane puncture samples; and assaying of urinary bacterial antigens.^2,3^ When pneumonia is associated with pleural effusion or empyema, the identification of its causative infectious agent can be done by culturing a pleural effusion sample.^4^

In our city, *Streptococcus pneumoniae*, followed by *Haemophilus influenzae* and *Staphylococcus aureus*, in this order, are the bacterial causative agents most frequently involved as etiologies for parapneumonic pleural effusions.^4-6^ These patients often have a good clinical outcome when treated with antibiotics and thoracocentesis.^7,8^ Some of them, however, have an unsatisfactory clinical course, despite these clinical measures. Surgical interventions are often indicated for the resolution of such cases. The surgical alternatives are chest tube underwater seal drainage, thoracoscopy, decortication and thoracostomy.^9^

The aim of the present study was to assess the etiology of complicated parapneumonic pleural effusions that cannot be resolved by clinical therapy alone and need surgical procedures to accomplish this task.

## PATIENTS AND METHODS

A retrospective study was conducted based on an analysis of 4,000 files on children hospitalized with pneumonia at the university hospital of the University of São Paulo from November 1986 until November 1996. Parapneumonic pleural effusion was a complication in 14.65% (586) of these patients, of whom 80.37% (471) followed a satisfactory clinical course using clinical therapy only (antibiotics and thoracocentesis).

Surgical treatment was considered in the remaining cases, in which clinical therapy was not effective. The clinical treatment was deemed to have failed when there was persistence of the septic state even with the use of adequate antibiotic therapy; recurrence of extensive effusions, requiring multiple thoracocentesis; septation or loculation inside the effusions; or pulmonary incarceration. In accordance with these criteria, 115 children needed a surgical procedure, during 117 hospitalization periods (two patients had a second episode of pneumonia complicated by pleural effusion during the period of study) and were included in this study.

## RESULTS

In our series, there were 58 female and 57 males, among whom there were 68 white and 47 black patients. One of our patients was a neonate, and there were 66 infants, 37 preschool and 11 school-age children. Concerning the nutritional status, we found that 89 were eutrophic, 23 had first degree malnutrition and three had second degree malnutrition, in accordance with the tables from the National Center for Health and Statistics Tables^10^ and the Gómez weight and height classification^11^ for each patient's age.

Assessment of the vaccination coverage revealed that 109 children had a complete immunization record, five had an incomplete one and, in one case, the neonate subject, immunization had not yet been begun.

Investigation of the birth conditions showed that 66 children were delivered normally, 40 were delivered surgically, forceps were used in eight cases, and the data was unavailable in one case. The birth weight was normal for 88 children, ten were underweight and, in 17 cases, the mothers were unable to give precise information.

The most frequently found previous morbid conditions were bronchopneumonia in 25 patients, bronchospasm in 20, acute otitis media in 16 and passive smoking in 10. Five children were declared to be attending daycare establishments.

The symptoms most frequently found were fever in 111 children, dyspnea in 96, cough in 90, prostration in 60 and wheezing in 46 patients. The radiological findings from these children are presented in Table l.

**Table 1:** Radiological findings from 117 parapneumonic pleural empyema cases

Parenchymatous disease	Number of patients	Pleural disease	Number of patients
Local pneumonia	16 (13.7%)	Small effusion	17 (14.5%)
Unilateral bronchopneumonia	94 (80.3%)	Medium-sized effusion	76 (65%)
Bilateral bronchopneumonia	7 (6%)	Complete hemithorax veiling	24 (20.5%)
**Total**	**117 (100%)**	**Total**	**117 (100%)**

Pleural effusions that only blurred the costophrenic angle were considered to be minimal and those that reached approximately the lower half of the hemithorax area were described as medium-sized. Sixty-one children had already taken antibiotics previously, and in 56 cases there was no mention of its use prior to hospitalization.

Table 2 presents the results from pleural effusion bacterioscopy and also from pleural effusion culturing. Of the 117 cultures, 54 were found to be positive, and 63 negative for microbial agents.

**Table 2:** Bacterioscopy and culturing results from pleural effusions in 117 parapneumonic pleural empyema cases

Bacterioscopy		Culture	
**Bacteria**	**Number**	**Positive**	**Negative**
Absent	47 (40.2%)	8 (6.9%)	39 (33.3%)
Present	70 (59.8%)	46 (39.3%)	24 (20.5%)
**Total**	**117 (100%)**	**54 (46.2%)**	**63 (53.8%)**

In seven patients, the causative agent was found via blood culturing but not via pleural effusion culturing. In eight patients, the agent was found in both the blood culture and the pleural effusion culture. Fifteen blood cultures were positive and 102 were negative for microbial agents, out of a total of 117.

We successfully isolated 73 agents: 58 from pleural effusion cultures and 15 from blood cultures. Two different agents were found in the pleural effusion cultures from four patients and, among those eight patients for whom both pleural effusion culturing and blood culturing were found to be positive, there were five patients with two different etiological agents. Table 3 shows the agents found, while Table 4 shows the agents found, according to the different age groups.

**Table 3:** Agents encountered in pleural effusions and blood cultures from 117 parapneumonic pleural empyema cases

Infection agent	Total	Pleural effusion	Blood culture
*Streptococcus pneumoniae*	45 (61.6%)	34 (58.6%)	11 (73.4%)
*Staphylococcus aureus*	11 (15%)	9 (15.5%)	2 (13.3%)
*Haemophilus influenza*	4 (5.5%)	2 (3.5%)	2 (13.3%)
*Streptococcus pyogenes*	4 (5.5%)	4 (6.9%)	-
*Escherichia coli*	3 (4.1%)	3 (5.2%)	-
*Enterococcus sp.*	2 (2.7%)	2 (3.5%)	-
*Klebsiella pneumoniae*	1 (1.4%)	1 (1.7%)	-
*Gram-negative bacilli*	1 (1.4%)	1 (1.7%)	-
*Pseudomonas aeruginosa*	1 (1.4%)	1 (1.7%)	-
*Proteus mirabilis*	1 (1.4%)	1 (1.7%)	-
**Total**	**73 (100%)**	**58 (100%)**	**15 (100%)**

**Table 4:** Agents encountered in different age groups, among 115 children with 117 cases of parapneumonic pleural empyema

**Age** Agent	**Infants** (1 month - 2 years)	**Preschool** (2 - 5 years)	**School-age children** (> 5 years)	**Total**
*Streptococcus pneumoniae*	16 (47.1%)	18 (72%)	5 (71.4%)	39 (59.1%)
*Staphylococcus aureus*	8 (23.6%)	1 (4%)	1 (14.3%)	10 (15.2%)
*Haemophilus influenza*	3 (8.8%)	1 (4%)	-	4 (6.1%)
*Streptococcus pyogenes*	2 (5.9%)	2 (8%)	-	4 (6.1%)
*Escherichia coli*	2 (5.9%)	-	1 (14.3%)	3 (4.5%)
*Enterococcus sp.*	1 (2.9%)	1 (4%)	-	2 (3%)
*Klebsiella pneumoniae*	-	1 (4%)	-	1 (1.5%)
*Gram-negative bacilli*	1 (2.9%)	-	-	1 (1.5%)
*Pseudomonas aeruginosa*	-	1 (4%)	-	1 (1.5%)
*Proteus mirabilis*	1 (2.9%)	-	-	1 (1.5%)
**Total**	**34 (100%)**	**25 (100%)**	**7 (100%)**	**66 (100%)**

*one neonate patient was excluded because the culturing was negative.

**Note:** *Since Streptococcus pneumoniae was isolated in 6 patients from pleural effusion culturing and blood culturing, and Staphylococcus aureus was isolated in one patient from two sites, there were 66 agents in our series, overall.*

The clinical therapy included 265 antibiotic prescriptions. This consisted of a single drug for 32 patients, two drugs for 36, three drugs for 38, four drugs for eight and five drugs for three patients (Table 5).

**Table 5:** Antibiotic prescriptions in 117 parapneumonic pleural empyema cases

Antibiotic	Number of prescriptions
Penicillin	76 (28.7%)
Chloramphenicol	67 (25.3%)
Oxacillin	65 (24.5%)
Vancomycin	18 (6.8%)
Amikacin	9 (3.4%)
Ceftriaxone	9 (3.4%)
Others	21 (7.9%)
**Total**	**265 (100%)**

## DISCUSSION

The vaccination coverage and adherence to child immunization programs of the population within the university hospital catchment area were very high. Almost all children in this study were found to have a complete immunization record. This finding correlates well with other authors’ results,^1,12^ in which similar high proportions of immunized children were found, although no correlations were found between immunization status and the socioeconomic conditions in São Paulo city. These authors^10-12^ considered birth weight to be a representative index for the quality of prenatal care and for the mother's and family's living conditions. Only 10 children in our series had low birth weight, and all of these had had previous acute respiratory infection. This is in accordance with other authors’ data,^12-14^ in which birth weight was identified as the most important factor associated with morbidity and mortality among infants.

The majority of the children in the present study came from the lower social strata. Nevertheless, they were in a good nutritional state. The patients with pleural effusions were predominantly infants. This finding is similar to data published by other authors.^4,7,13^ The hospitalizations occurred most frequently during the southern hemisphere spring (mostly in November). We believe that pleural empyema would be expected to occur more frequently during the months immediately following the peak incidence period for pneumonia.

In the present series, 82% of the patients were found to be dyspneic upon admission to hospital, and 51.3% were considered prostrated. This leads us to conclude that pleural effusions have a high potential for discomfort.^12-14^

Some authors^12-14^ have considered that the most important risk factors for pulmonary disease among children are parents with a low regular education level; large numbers of people living in the same house; very young mother; child attending a daycare establishment; or previous episodes of pulmonary disease or bronchospasm. In our series, it was remarkable that 57.3% of the children had a previous history of acute respiratory infection or bronchospastic episodes.

We found that 52.13% of the patients had made use of antibiotics. No measurement was made of either the concentration of antimicrobial agents in the pleural effusions or the bactericidal power of the serum. We did, however, observe that antibiotic therapy interfered with culture positivity. Out of 24 patients who had positive bacterioscopy and negative pleural effusion culture, 20 had received previous antibiotic therapy, and bacterioscopy performed on the other four showed that they had Gram-negative bacilli, probably *Haemophilus*. This agent may not have grown in cultures because of difficulties peculiar to the cultivation method for this agent. Eight patients had positive blood cultures for microbial agents, even though the pleural effusion being negative.

The identification of the etiological agents for pneumonia on the basis of pleural effusion culturing is believed to be sufficiently reliable, although there is a tendency for some bacteria to produce more pleural effusions than others.^15-17^ It has been shown that pleural effusion occurs in 10% of pneumonia cases caused by *Streptococcus pneumoniae*, 35% caused by anaerobes and 50% caused by *Streptococcus pyogenes*.^3,4^

Many authors^18,19^ have concluded that the obtaining of bronchial tree secretion samples by means of bronchoscopy is not a reliable alternative, because of possible contamination of the bronchoscope by oropharyngeal germs. Also, it has been demonstrated that there is no correlation between the germs found in the oropharynx and those found at the pneumonic focus.^20^ For these reasons, bronchoscopy was not used on our patients.

The causative agent for pleural effusions has been identified in 50 to 55.7% of the cases, in most series.^4-6^ Pleural effusion culture positivity varies according to its appearance: rates of 77% for purulent effusions and 26% for serous effusions have been found.

In the same way as for other authors^4-6^ who studied the etiological agents for pneumonia in a Brazilian setting, we also found predominance of *Streptococcus pneumoniae*. The participation of *Haemophilus influenzae* in the etiology of pulmonary disease was of little importance in our series. However, it is possible to infer a more important role for this agent because of the bacterioscopy findings in pleural effusion cases, technical difficulties related to laboratory culturing and the high responsiveness to therapy of patients with negative cultures.^21^

We observed that in 208 (78.4%) of the antibiotic prescriptions, out of the 265 needed for treating these patients’ pneumonia, the pleural and parenchymatous disease were resolved using crystalline penicillin, chloramphenicol and oxacillin. This signifies that, despite the high pathogenicity and infectivity of the microbial agents, there was no correlation with multiresistance. The greater severity and worse outcome of our cases for which surgical intervention was necessary may have been due to the antigenic and surfactant characteristics of these agents. More likely, these children were somehow immunoincompetent. Possibly, these factors determined worse outcome because of more intensive capillary permeability change, and more extensive protein extravasation into the alveolar space.

Nonetheless, although these patients had a worse outcome and needed surgical treatment to cure the pleural empyema, the etiological agents were the same as those associated with empyema resolved by clinical therapy alone.

## CONCLUSION

From our verification of the bacteriological profile of the extensive severe pneumonia cases that required surgical intervention, we concluded that this profile resembled what has been described for non-complicated pneumonia cases, except for the increased frequency of *Staphylococcus aureus* in the present study. The worse clinical evolution of these children must be due to the virulence of the causative agent or to matters relating to their immunological capacity. Future studies will be required for such investigations.
